# Post-activation performance enhancement in resisted sprinting: effects of different loads and rest intervals on 100-m sprint segments

**DOI:** 10.3389/fphys.2025.1544291

**Published:** 2025-08-12

**Authors:** Weixiong Chen, Dexing Qian, Huaichuan Zhang, Yanfei Shen

**Affiliations:** ^1^ School of Sports Engineering, Beijing Sport University, Beijing, China; ^2^ China Athletics College, Beijing Sport University, Beijing, China

**Keywords:** post-activation potentiation enhancement, sprint, load, rest interval, resisted sprint training

## Abstract

This study aimed to investigate the acute effects of resisted sprint-induced post-activation potentiation enhancement (PAPE) on 100-m sprint performance under three loading conditions (5%, 10%, and 15% body weight) and three rest intervals (4, 8, and 12 min), with a focus on segmental performance (0–30 m, 30–60 m, and 60–100 m). In a randomized crossover design, ten male college sprinters (age: 19.2 ± 1.5 years; 100-m personal best: 11.31 ± 0.30 s) performed nine experimental tests over 19 days. Each trial included a 40-m resisted sprint using the motorized Jueying™ system (Beijing Sport University) followed by a 100-m sprint under one of the nine load-rest combinations. Sprint times were measured via SmartSpeed™ timing gates. The 10% BW load with an 8-min rest interval elicited the greatest PAPE effect, significantly improving performance in the 0–30 m ( Δ = 0.192 s, p < 0.01; Cohen’s d = 1.66) and 30–60 m ( Δ = 0.154 s, p < 0.05; d = 1.29) segments. However, no significant improvements were observed in the 60–100 m segment. The loads of 5% and 15% BW showed smaller or inconsistent effects in the rest intervals.

## 1 Introduction

In the highly anticipated Olympic Games, the 100-m sprint has become the focal point of track and field events, emphasizing athletes’ ability to achieve rapid acceleration and reach maximum speed over extremely short distances. The 100-m sprint comprises three primary phases: the acceleration phase, the maximum velocity phase, and the deceleration phase (Jones et al., 2009; Mero et al., 1992). Researchers have examined various sprint parameters, including split times, step length, step time, and ground contact time, within each phase (Chang et al., 2024; Colyer et al., 2018; Morin et al., 2012).

The acceleration after the start is a key technique in the 100-m dash. Importantly, the quality of this phase directly affects the maximum speed of the athlete (Harland and Steele, 1997; Slawinski et al., 2010). Top-level sprinters typically reach their maximum speed between 50 and 70 m. Therefore, the acceleration phase for these athletes can last longer and represent more than 30% of the total performance (Maćkała et al., 2015; Seidl et al., 2021). In contrast, non-elite athletes tend to reach their maximum speed earlier, resulting in a shorter acceleration phase (Haugen et al., 2019). Resisted sprint preactivation is a vital method to improve the ability of athletes to accelerate after the start by improving neuromuscular recruitment and increasing ground reaction forces during the start phase, allowing athletes to reach maximum or submaximum speeds from a stationary position in a shorter period of time (Faccioni, 1994; Clark et al., 2010). Resistance sprint exercises take the form of pulling a sled, tires or parachute, or using advanced equipment like the 1080Sprint™(1,080 Motion, Sweden) and DynaSpeed™(Ergotest Technology AS, Norway), which provide constant resistance during acceleration (Haugen et al., 2019; Matusiński et al., 2021a; Cross et al., 2018; Mangine et al., 2018).

Resisted sprint training yields significant effects in both long-term adaptations and acute changes. Long-term adaptations occur in trained athletes after several weeks of progressive training, leading to sustained improvements in performance. In contrast, acute changes refer to the immediate physiological responses following a single pre-activation session, where increased resistance enhances muscle activation and subsequently improves performance (Ross et al., 2001).

These acute changes are closely related to the concept of postactivation potentiation enhancement (PAPE). PAPE refers to the short-term increase in muscle force and power output following high-intensity exercise, such as resisted sprints (Prieske et al., 2020; Blaževich and Babault, 2019). Therefore, incorporating resisted sprints into a pre-activation regimen can be an effective strategy to harness the benefits of PAPE and achieve immediate performance enhancements. There has been considerable research on the effectiveness of resisted pre-activation to improve sprint performance, with loads ranging from 5% to 150% of body weight (BW) (Matusiński et al., 2021a; Mangine et al., 2018; Godwin et al., 2023; Winwood et al., 2016; Seitz et al., 2017; Van Den Tillaar and Von Heimburg, 2017; Alcaraz et al., 2008; Petrakos et al., 2016). However, selecting the optimal load for advanced training equipment to maximize the benefits of PAPE remains a challenge. Selecting optimal resistance loads requires an individualized strategy that integrates athlete-specific profiles with periodization goals. A wealth of research, including several notable studies, has investigated this challenge and concluded that a resisted sprint with 10% BW load can effectively induce a potentiating effect on subsequent sprint performance. Specifically, this load has been found to improve 20-m flying start sprint performance in elite female sprinters, demonstrating the potential for customized load selection to optimize PAPE outcomes (Matusiński et al., 2021a). Furthermore, external loads between 8% and 13% of BW appear to be optimal for simultaneously enhancing both power and sprint speed (Matusiński et al., 2021b). Furthermore, they concluded that resisted sprint pre-activation with a load of 10% BW improves sprinting speed over 50 m in elite male and female sprinters (Matusiński et al., 2022). However, it remains uncertain whether performance enhancement might also be observed over longer distances or during different phases of the sprint. Elite athletes exhibit extended acceleration phases during the start. Resisted sprints of 10–30 m may present limitations.

Research indicates that stronger individuals tend to exhibit more pronounced PAPE responses. This is due to their greater muscle mass and neuromuscular efficiency, allowing them to generate higher levels of force following a PAPE protocol. For instance, stronger athletes have shown significant improvements in performance metrics such as countermovement jump (CMJ) height and sprint times following high-intensity conditioning activities (Sa et al., 2020; Guo et al., 2022).

Given the established relationship between strength and PAPE, we hypothesized that athletes with superior 100-m sprint performance, who are likely to possess greater strength, would exhibit more notable improvements in the initial five steps of their sprint due to the PAPE effect. During the optimal rest period, when the PAPE is anticipated to be most effective, the performance in the initial five steps of a sprint was analyzed (Brink et al., 2022; Maroto-Izquierdo et al., 2020). By focusing on this optimal rest interval across all load conditions, we aimed to standardize the conditions and directly assess the impact of varying loads on sprint kinematics without the confounding influence of different recovery times. This approach allowed for a targeted investigation into how the potentiation effect, at its presumed peak, interacts with different levels of resistance to influence sprint performance.

## 2 Materials and methods

### 2.1 Participants

Ten male collegiate sprinters (age: 19.2 ± 1.5 years; body mass: 74.5 ± 10.1 kg; height: 181.3 ± 5.8 cm; 100-m personal best: 11.305 ± 0.297 s) classified as Tier 3: Trained/Developmental athletes according to McKay’s classification framework participated in this study. Inclusion criteria required (Jones et al., 2009): ≥ 2 years of resisted sprint training experience (Mero et al., 1992), no prior use of the Jueying™ system, and (Chang et al., 2024) absence of musculoskeletal injuries or cardiovascular abnormalities. Participants maintained consistent training footwear (spikes) and refrained from maximal training and stimulants for 48 h before testing. The study protocol was approved by Beijing Sport University’s Ethics Review Board (No. 2024037H), with written informed consent obtained from all participants.

### 2.2 Experimental design

We employed a randomized, counterbalanced crossover design with three experimental factors: resistance loads (5%, 10%, and 15% of body mass), recovery intervals (4, 8, and 12 min), and sprint segments (0–30 m, 30–60 m, and 60–100 m). Each participant completed nine post-tests in total, corresponding to all combinations of load and rest conditions. To minimize order effects, the sequence of post-tests was randomized for each individual, with both the resistance load and recovery interval randomly assigned in each trial. The 19-day protocol was conducted at consistent times of day ( ± 0.5 h) in Beijing Sport University’s indoor track facility, with sessions separated by ≥ 48 h to minimize fatigue accumulation.

Resistance application was standardized using the motorized Jueying™ system (School of Sports Engineering, Beijing Sport University), which delivers velocity-dependent loads via a cable-driven mechanism (Hanran et al., 2024). Based on established dose-response relationships in post-activation performance enhancement (PAPE) (Matusiński et al., 2021a; Godwin et al., 2023; Brink et al., 2022), three relative loads were selected to systematically compare fatigue-potentiation dynamics. Recovery intervals were strategically chosen to capture the temporal transition from fatigue dominance (4 min) to PAPE predominance (8–12 min), consistent with established recovery kinetics (Boullosa et al., 2011; Chen et al., 2023; Kilduff et al., 2011).

### 2.3 Activation protocol

#### 2.3.1 Equipment configuration

**FIGURE 1:**
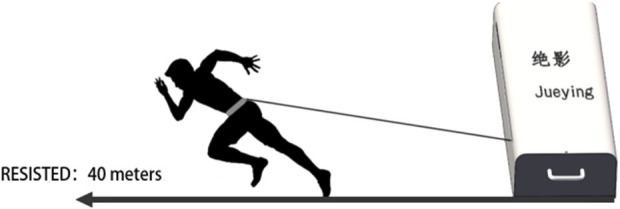
Jueying system for sprint experiments.

The motorized Jueying™ system was positioned 0.5 m behind the starting line, delivering velocity-dependent resistance through a cable-driven mechanism, as shown in Figure 1. Sprint times were recorded using the SmartSpeed™ timing system (VALD Performance, Australia), calibrated at 0, 30, 60, and 100 m positions following manufacturer specifications (Bond et al., 2017; Larson and Noonan, 2014).

#### 2.3.2 Testing procedures

Session 1 (Baseline) consisted of anthropometric measurements followed by three maximal 100-m sprints, with the best performance retained for subsequent analysis. Sessions 2–10 (Intervention) comprised four sequential components (Jones et al., 2009): standardized warm-up protocol (20 min general preparation +10 min sprint-specific drills), (Mero et al., 1992),, 40-m resisted sprint under assigned load condition (Chang et al., 2024), passive recovery during the prescribed interval, and (Colyer et al., 2018) 100-m maximal sprint performance evaluation. To control for learning effects, condition order was randomized (Kang et al., 2008; Netz et al., 2019), with all sprints initiated from a standardized three-point stance 0.3 m behind the starting line (Mangine et al., 2018; Seitz et al., 2017).

### 2.4 Dependent variables

Primary outcomes included split times for three acceleration phases (0–30 m, 30–60 m, and 60–100 m) and total 100-m sprint time. These measures were selected based on their established sensitivity to resisted sprint training interventions (Bond et al., 2017; Cohen, 2013).

### 2.5 Statistical analysis

Statistical power was determined *a priori* using G*Power 3.1, indicating a minimum sample size of 7 participants for repeated-measures ANOVA with effect size = 0.4, power = 0.8, and α=0.05 . Our final sample (n = 10) exceeded this requirement. Data analysis employed three-way repeated measures ANOVA (load × interval × distance) with Greenhouse-Geisser corrections for sphericity violations. Post-hoc comparisons used Bonferroni-adjusted α levels (0.05) with effect size quantification through partial η ^2^ for ANOVA effects and Cohen’s d for pairwise comparisons (Cohen, 2013). All analyses were conducted in SPSS 25.0 (IBM Corp.), with normality confirmed via Shapiro-Wilk tests (p > 0.05) and results reported as mean ± SD.

## 3 Results

The Shapiro-Wilk tests indicated that the sprint time data conformed to a normal distribution, permitting the use of parametric statistical analyses. Subsequent repeated-measures analyses were conducted to assess the impact of the intervention across various segments of the sprint. A linear trend analysis revealed no significant change in baseline sprint times across sessions (p > 0.05), suggesting no learning or fatigue effect over time. The pairwise comparison results, which provide a detailed examination of the differences between each condition, are presented in Tables 1–4.

**TABLE 1:** Effects of different loads inducing PAPE on 0–30 meters performance.

Resistance load	Rest interval	Time (s)	Diff. To baseline (s)	Effect Size (95%CI)	Performance outcome
Baseline	—	4.193 ± 0.114	—	—	—
5% BW	4min	4.095 ± 0.122	0.098 ± 0.023	0.83 (0.59–1.07)	improved
	8min	4.060 ± 0.124	0.133 ± 0.022^*^	1.12 (0.88–1.36)	improved
	12min	4.138 ± 0.134	0.054 ± 0.013	0.44 (0.17–0.7)	worsened
10% BW	4min	4.042 ± 0.135	0.150 ± 0.020^*^	1.20 (0.94–1.47)	improved
	8min	4.001 ± 0.117	0.192 ± 0.022^*^	1.66 (1.44–1.89)	improved
	12min	4.081 ± 0.138	0.111 ± 0.022^*^	0.88 (0.61–1.15)	improved
15% BW	4min	4.076 ± 0.125	0.117 ± 0.019^*^	0.98 (0.74–1.22)	improved
	8min	4.080 ± 0.143	0.113 ± 0.018^*^	0.87 (0.59–1.15)	improved
	12min	4.121 ± 0.110	0.071 ± 0.018	0.64 (0.24–0.68)	no change

*: p < 0.05.

**TABLE 2:** Effects of different loads inducing PAPE on 30–60 meters performance.

Resistance load	Rest interval	Time (s)	Diff. To baseline (s)	Effect Size (95%CI)	Performance outcome
Baseline	—	3.305 ± 0.126	—	—	—
5% BW	4min	3.231 ± 0.125	0.074 ± 0.014^*^	0.59 (0.35–0.84)	no change
	8min	3.188 ± 0.125	0.118 ± 0.017^*^	0.94 (0.69–1.14)	improved
	12min	3.253 ± 0.141	0.053 ± 0.024	0.39 (0.12–0.67)	worsened
10% BW	4min	3.183 ± 0.125	0.122 ± 0.021^*^	0.97 (0.73–1.22)	improved
	8min	3.152 ± 0.111	0.154 ± 0.017^*^	1.29 (1.07–1.51)	improved
	12min	3.239 ± 0.130	0.067 ± 0.019	0.52 (0.26–0.77)	no change
15% BW	4min	3.209 ± 0.110	0.096 ± 0.017^*^	0.81 (0.60–1.03)	improved
	8min	3.209 ± 0.142	0.096 ± 0.019^*^	0.72 (0.44–1.00)	no change
	12min	3.250 ± 0.113	0.055 ± 0.021	0.46 (0.24–0.68)	worsened

p<0.05 .

**TABLE 3:** Effects of different loads inducing PAPE on 60–100 meters performance.

Resistance load	Rest interval	Time (s)	Diff. To baseline (s)	Effect Size (95%CI)	Performance outcome
Baseline	—	4.535 ± 0.181	—	—	—
5% BW	4min	4.419 ± 0.206	0.116 ± 0.046	0.60 (0.19–1.00)	no change
	8min	4.389 ± 0.214	0.147 ± 0.061	0.74 (0.32–1.16)	no change
	12min	4.434 ± 0.165	0.102 ± 0.051	0.59 (0.26–0.91)	no change
10% BW	4min	4.428 ± 0.193	0.107 ± 0.039	0.57 (0.19–0.95)	no change
	8min	4.393 ± 0.188	0.143 ± 0.038	0.77 (0.41–1.14)	no change
	12min	4.466 ± 0.198	0.010 ± 0.024	0.37 (-0.02–0.75)	worsened
15% BW	4min	4.450 ± 0.232	0.085 ± 0.039	0.41 (-0.04–0.86)	worsened
	8min	4.412 ± 0.207	0.123 ± 0.054	0.63 (0.23–1.04)	no change
	12min	4.516 ± 0.202	0.019 ± 0.029	0.10 (-0.30–0.49)	worsened

p<0.05 .

**TABLE 4:** Effects of different loads inducing PAPE on 0–100 meters performance.

Resistance load	Rest interval	Time (s)	Diff. to baseline (s)	Effect size (95%CI)	Performance outcome
Baseline	—	12.033 ± 0.385	—	—	—
5% BW	4 min	11.745 ± 0.435	0.288 ± 0.059*	0.70 (-0.15-1.55)	no change
	8 min	11.636 ± 0.393	0.397 ± 0.056*	1.02 (0.25-1.79)	improved
	12 min	11.826 ± 0.357	0.207 ± 0.036*	0.56 (-0.14-1.26)	no change
10% BW	4 min	11.654 ± 0.412*	0.380 ± 0.052*	0.95 (0.15-1.76)	improved
	8 min	11.545 ± 0.385	0.488 ± 0.059*	1.27 (0.51-2.02)	improved
	12 min	11.786 ± 0.429	0.247 ± 0.044*	0.61 (-0.23-1.45)	no change
15% BW	4 min	11.735 ± 0.413	0.298 ± 0.048*	0.75 (-0.06-1.56)	no change
	8 min	11.693 ± 0.427	0.340 ± 0.063*	0.84 (0.00-1.67)	improved
	12 min	11.888 ± 0.389	0.145 ± 0.037	0.38 (-0.39-1.14)	worsened

*: p < 0.05.

In the 0–30 m segment, the 10% load with an 8-min rest produced the greatest time reduction of 0.192 s compared to baseline (from 4.193 ± 0.114 s to 4.001 ± 0.117 s), equating to a 4.64% improvement and a large effect size (Cohen’s d = 1.66). Similarly, in the 30–60 m segment, the same condition resulted in a 0.154-s improvement (from 3.305 ± 0.126 s to 3.152 ± 0.111 s), or 4.67%, with a large effect size of 1.29. Although improvements in the 60–100-m segment were not statistically significant, a moderate performance improvement of 0.143 s (3.15%, d = 0.77) was still observed under this optimal condition.

Throughout the 0–100 m sprint, condition 10% load with 8 min yielded a total time improvement of 0.488 s over baseline (from 12.033 ± 0.385 s to 11.545 ± 0.385 s), representing a 4. 06% improvement with a large effect size (Cohen’s d = 1.27). In contrast, the 5% and 15% load conditions showed smaller and more inconsistent benefits. For example, the 5% load at 8 min improved the total time by 0.397 s (3.30%, d = 1.02), and the 15% load at 8 min resulted in a 0.340 s reduction (2. 83%, d = 0.84), both less than the effect seen with the 10% load.

These findings suggest that the PAPE effect is phase specific, mainly benefiting the acceleration (0–30 m) and early transition (30–60 m) phases, where neural and muscular responsiveness to preactivation is highest. The lack of significant enhancement in the 60–100-m segment reinforces the notion that PAPE exerts less influence during maximal velocity maintenance, which is more dependent on elastic energy return and technique.

In conclusion, the present study identifies the 10% body weight resistance with an 8-min rest interval as the most effective condition for eliciting acute performance gains through PAPE, with time reductions up to 4.6% in acceleration, 4.7% in transition, and 4.1% overall, thereby providing a robust, data-driven protocol for sprint performance optimization.

## 4 Discussion

The current investigation provides novel evidence that resisted sprint pre-activation using motorized resistance systems can acutely enhance sprint performance when implemented with precise load and recovery parameters. Our central finding–the 10% BW load combined with an 8-min recovery interval eliciting significant acceleration-phase improvements–advances understanding of post-activation performance enhancement (PAPE) in sprint contexts (Matusiński et al., 2021a; Li et al., 2024). These results align with emerging research on velocity-dependent resistance training while challenging conventional paradigms regarding optimal resisted sprint duration (Faccioni, 1994; Mangine et al., 2018).

The observed superiority of 10% BW over lighter (5% BW) and heavier (15% BW) loads corroborates the inverted-U relationship between mechanical stimulus and PAPE responsiveness (Godwin et al., 2023; Brink et al., 2022). This phenomenon likely reflects two competing mechanisms: insufficient neural recruitment at lower intensities versus excessive fatigue accumulation at higher loads (Boullosa et al., 2011). Notably, the 40-m resisted sprint distance in our protocol contrasts with traditional 20–30 m recommendations (Clark et al., 2010), suggesting modern motorized systems may extend effective PAPE stimulus ranges through precise resistance modulation (Hanran et al., 2024). Unlike sled resistance, which can vary due to surface friction and running technique, motorized resistance provides consistent and precisely controlled load across the sprint distance. This allows for finer modulation of resistance intensity and minimizes variability between trials, enhancing the reproducibility of training stimuli and PAPE responses. This technological advancement enables athletes to accumulate greater impulse while maintaining movement velocity profiles–a critical factor absent in sled-based methodologies (Sa et al., 2020).

Phase-specific performance patterns revealed fundamental biomechanical insights. The 0–30m and 30–60 m improvements (1.8% and 1.2% respectively) versus negligible 60–100 m changes (−0.3%) underscore PAPE’s preferential impact on acceleration mechanics (Prieske et al., 2018). This dichotomy aligns with electromyographic evidence demonstrating enhanced activation of key agonist muscles—including the psoas major, gluteus maximus, hamstrings, gluteus medius, and ankle plantarflexors—during the initial acceleration phase, with markedly reduced activation observed at maximal velocity (Li et al., 2024). From an energy systems perspective, the phosphocreatine resynthesis window (4–8 min) may synergize with PAPE’s neural mechanisms during early sprint phases (Chen et al., 2023), whereas late-phase velocity maintenance relies more on elastic energy utilization–a system less influenced by acute resistance priming (Kilduff et al., 2011).

Methodological considerations warrant particular emphasis. The discrepancy between initial pairwise comparisons and conservative post hoc results highlights the importance of statistical rigor in multi-factorial PAPE studies (Cohen, 2013). While traditional resisted sprint research often employs uncorrected comparisons (Faccioni, 1994), our Bonferroni-adjusted approach revealed that only the 10% BW condition maintained significance–a finding with critical implications for coaching practice (Netz et al., 2019). This statistical conservatism proves essential when balancing type I error risks against the practical need for actionable training prescriptions.

Several limitations contextualize these findings. The absence of synchronized kinetic data restricts mechanistic interpretation of force-velocity adaptations. Furthermore, the lack of neuromuscular data (e.g., EMG), force–velocity profiling, and kinematic analyses, which limits our understanding of the underlying mechanisms. Furthermore, we clarify that the sample consisted exclusively of male athletes, so the findings cannot be directly generalized to female populations.

## 5 Conclusion

This study shows that a 10% BW load with an 8-min rest interval effectively enhances sprint performance, particularly in the 0–30 and 30–60 m segments. While resisted sprint pre-activation significantly improves acceleration, its impact on maintaining speed in the 60–100 m segment is limited.

## Data Availability

The datasets presented in this study can be found in online repositories. The names of the repository/repositories and accession number(s) can be found in the article/Supplementary Material.
