# Supplementary material for: Post-activation performance enhancement in resisted sprinting: effects of different loads and rest intervals on 100-m sprint segments
Source: Front Physiol. 2025 Aug 12;16:1544291. doi: 10.3389/fphys.2025.1544291 (PMC12378636; doi:10.3389/fphys.2025.1544291)
Supplement: Supplementary file 1 [file DataSheet1.pdf]

post-test time

0-30m	30-60m	60-100m	0-100m
4.165	3.193	4.330	11.688
3.984	3.120	4.386	11.490
4.297	3.232	4.590	12.119
4.300	3.348	4.692	12.340
4.282	3.360	4.524	12.166
4.262	3.373	4.549	12.184
4.194	3.313	4.587	12.094
4.037	3.176	4.222	11.435
4.122	3.395	4.633	12.150
4.282	3.544	4.840	12.666

5% of BW with 4-minute rest

0-30m	30-60m	60-100m	0-100m
4.031	3.160	4.390	11.581
3.905	3.112	4.168	11.185
4.061	3.166	4.161	11.388
4.288	3.322	4.617	12.227
4.101	3.231	4.453	11.785
4.137	3.292	4.648	12.077
4.123	3.189	4.436	11.748
3.932	3.053	4.144	11.129
4.113	3.296	4.472	11.881
4.256	3.489	4.703	12.448

5% of BW with 8-minute rest

0-30m	30-60m	60-100m	0-100m
4.044	3.131	4.260	11.435
3.835	3.022	4.227	11.084
4.053	3.153	4.169	11.375
4.247	3.284	4.168	11.699
4.056	3.246	4.622	11.924
4.077	3.319	4.434	11.830
4.079	3.116	4.398	11.593
3.911	3.005	4.225	11.141
4.067	3.210	4.635	11.912
4.229	3.389	4.747	12.365

5% of BW with 12-minute rest

0-30m	30-60m	60-100m	0-100m
4.033	3.122	4.233	11.388
3.921	3.025	4.542	11.497
4.267	3.370	4.225	11.862
4.274	3.312	4.530	12.116
4.182	3.357	4.438	11.977
4.229	3.244	4.678	12.151
4.147	3.266	4.478	11.891
3.941	3.077	4.200	11.218
4.117	3.281	4.423	11.821
4.272	3.475	4.589	12.336

10% of BW with 4-minute rest

0-30m	30-60m	60-100m	0-100m
4.056	3.105	4.333	11.494
3.810	2.995	4.180	10.985
4.079	3.204	4.253	11.536
4.214	3.228	4.519	11.961
4.011	3.146	4.439	11.596
4.158	3.214	4.448	11.820
4.062	3.115	4.370	11.547
3.839	3.133	4.286	11.258
4.005	3.213	4.624	11.842
4.189	3.517	4.831	12.537

10% of BW with 8-minute rest

0-30m	30-60m	60-100m	0-100m
4.007	3.082	4.292	11.381
3.844	3.034	4.235	11.113
3.980	3.018	4.166	11.164
4.185	3.229	4.426	11.840
3.990	3.166	4.404	11.560
4.052	3.202	4.438	11.692
3.995	3.196	4.431	11.622
3.817	3.063	4.175	11.055
3.973	3.139	4.595	11.707
4.163	3.389	4.764	12.316

10% of BW with 12-minute rest

0-30m	30-60m	60-100m	0-100m
4.088	3.180	4.307	11.575
3.852	3.106	4.290	11.248
4.088	3.180	4.334	11.602
4.286	3.306	4.670	12.262
4.057	3.213	4.474	11.744
4.171	3.371	4.462	12.004
4.109	3.288	4.492	11.889
3.932	3.028	4.170	11.130
3.970	3.235	4.662	11.867
4.261	3.481	4.797	12.539

15% of BW with 4-minute rest

0-30m	30-60m	60-100m	0-100m
4.106	3.149	4.247	11.502
3.863	3.092	4.266	11.221
4.127	3.210	4.220	11.557
4.262	3.275	4.614	12.151
4.104	3.171	4.387	11.662
4.078	3.263	4.407	11.748
4.055	3.174	4.638	11.867
3.961	3.057	4.200	11.218
3.957	3.253	4.636	11.846

4.242	3.448	4.886	12.576

15% of BW with 8-minute rest

0-30m	30-60m	60-100m	0-100m
4.100	3.089	4.412	11.601
3.853	3.050	4.207	11.110
4.104	3.140	4.094	11.338
4.271	3.309	4.571	12.151
4.190	3.139	4.580	11.909
4.116	3.296	4.591	12.003
4.078	3.303	4.368	11.749
3.897	3.031	4.168	11.096
3.939	3.259	4.413	11.611
4.251	3.475	4.720	12.360

15% of BW with 12-minute rest

0-30m	30-60m	60-100m	0-100m
4.088	3.193	4.237	11.518
3.964	3.103	4.289	11.356
4.154	3.207	4.523	11.884
4.240	3.373	4.558	12.171
4.177	3.264	4.574	12.015
4.168	3.373	4.738	12.279
4.190	3.279	4.553	12.022
4.026	3.075	4.244	11.345
3.949	3.222	4.618	11.789
4.256	3.412	4.830	12.498
